# 4-[(4-Meth­oxy­benzene­sulfonamido)­meth­yl]cyclo­hexane-1-carb­oxy­lic acid

**DOI:** 10.1107/S1600536811030650

**Published:** 2011-08-06

**Authors:** Muhammad Ashfaq, Samina Iram, Mehmet Akkurt, Islam Ullah Khan, Ghulam Mustafa, Muhammad Danish

**Affiliations:** a Department of Chemistry, University of Gujrat, H.H. Campus, Gujrat 50700, Pakistan; b Department of Physics, Faculty of Sciences, Erciyes University, 38039 Kayseri, Turkey; c Department of Chemistry, Government College University, Lahore 54000, Pakistan

## Abstract

In the title compound, C_15_H_21_NO_5_S, two crystallographically independent mol­ecules are linked into a dimer by a pair of N—H⋯O hydrogen bonds, forming an *R*_2_^2^(8) ring motif. In the crystal, mol­ecules are further linked by inter­molecular O—H⋯O hydrogen bonds into a two-dimensional network parallel to (012). Additional stabilization is provided by weak inter­molecular C—H⋯O hydrogen bonds.

## Related literature

For background to tranexamic acid (trans-4-(amino­meth­yl)cyclo­hexa­necarb­oxy­lic acid), see: Boylan *et al.* (1996 ▶); Khan *et al.* (2002 ▶); Nilsson (1980 ▶); Shah *et al.* (2010 ▶); Shahzadi *et al.* (2007 ▶); Svahn *et al.* (1986 ▶); Vavrova *et al.* (2005 ▶). For a related structure, see: Ashfaq *et al.* (2011 ▶). For hydrogen-bond motifs, see: Bernstein *et al.* (1995 ▶). For ring conformations, see: Cremer & Pople (1975 ▶).
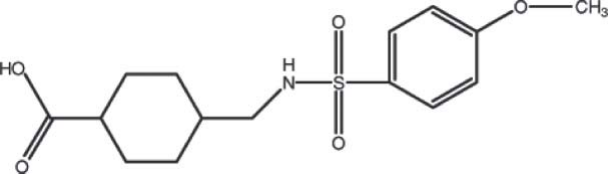


## Experimental

### 

#### Crystal data

C_15_H_21_NO_5_S
*M* *_r_* = 327.40
Triclinic, 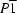

*a* = 5.9119 (3) Å
*b* = 10.7223 (7) Å
*c* = 26.6453 (15) Å
α = 79.736 (3)°
β = 89.813 (3)°
γ = 75.518 (3)°
*V* = 1607.67 (16) Å^3^
*Z* = 4
Mo *K*α radiation
μ = 0.22 mm^−1^
*T* = 296 K
0.25 × 0.19 × 0.14 mm

#### Data collection

Bruker APEXII CCD diffractometer
25440 measured reflections
5942 independent reflections
3973 reflections with *I* > 2σ(*I*)
*R*_int_ = 0.048

#### Refinement

*R*[*F* ^2^ > 2σ(*F* ^2^)] = 0.070
*wR*(*F* ^2^) = 0.218
*S* = 1.03
5942 reflections
412 parameters
5 restraints
H atoms treated by a mixture of independent and constrained refinement
Δρ_max_ = 0.92 e Å^−3^
Δρ_min_ = −0.36 e Å^−3^

### 

Data collection: *APEX2* (Bruker, 2007 ▶); cell refinement: *SAINT* (Bruker, 2007 ▶); data reduction: *SAINT*; program(s) used to solve structure: *SHELXS97* (Sheldrick, 2008 ▶); program(s) used to refine structure: *SHELXL97* (Sheldrick, 2008 ▶); molecular graphics: *ORTEP-3 for Windows* (Farrugia, 1997 ▶); software used to prepare material for publication: *WinGX* (Farrugia, 1999 ▶) and *PLATON* (Spek, 2009 ▶).

## Supplementary Material

Crystal structure: contains datablock(s) global, I. DOI: 10.1107/S1600536811030650/lh5293sup1.cif

Structure factors: contains datablock(s) I. DOI: 10.1107/S1600536811030650/lh5293Isup2.hkl

Supplementary material file. DOI: 10.1107/S1600536811030650/lh5293Isup3.cml

Additional supplementary materials:  crystallographic information; 3D view; checkCIF report

## Figures and Tables

**Table 1:** Hydrogen-bond geometry (Å, °)

*D*—H⋯*A*	*D*—H	H⋯*A*	*D*⋯*A*	*D*—H⋯*A*
N1—H1*N*⋯O8^i^	0.83 (4)	2.14 (3)	2.961 (4)	173 (3)
N2—H2*N*⋯O3^ii^	0.85 (3)	2.18 (3)	3.024 (4)	172 (3)
O4—H4*O*⋯O5^iii^	0.83 (5)	1.84 (5)	2.660 (5)	166 (6)
O10—H10*O*⋯O9^iv^	0.82 (7)	1.89 (8)	2.631 (8)	151 (7)
C16—H16*B*⋯O6^v^	0.96	2.59	3.378 (7)	139
C25—H25*A*⋯O3^ii^	0.97	2.54	3.450 (6)	157

Symmetry codes: (i) 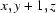; (ii) 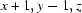; (iii) 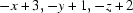; (iv) 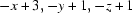; (v) 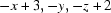.

**Fig. 1.**
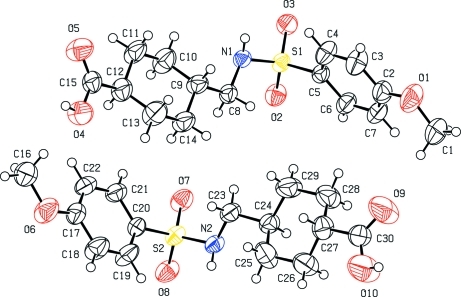
The two independent molecules in the asymmetric unit with displacement ellipsoids for non-H atoms drawn at the 50% probability level.

**Fig. 2.**
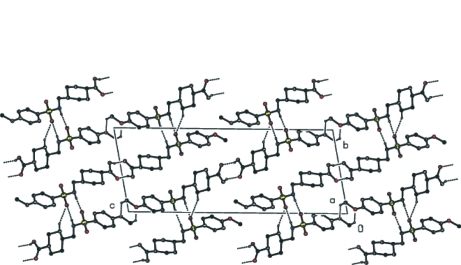
The packing and hydrogen bonding of viewed along the a axis. Hydrogen atoms not involved in hydrogen bonding have been omitted for clarity.

## supplementary crystallographic information

### Comment

Tranexamic acid or trans-4-(aminomethyl)cyclohexanecarboxylic acid, is a synthetic amino acid used commonly for curing abnormal bleeding in a variety of diseases (Boylan *et al.*, 1996; Nilsson, 1980). A number of scientists derivatized this drug, evaluated the activity and found most derivatives were superior to the parent drug (Svahn *et al.*, 1986; Khan *et al.*, 2002; Vavrova *et al.*, 2005; Shahzadi *et al.*, 2007; Shah *et al.*, 2010). We have recently prepared a derivative of this drug and reported the crystal structure (Ashfaq *et al.*, 2011).

Fig. 1 shows the two crystallographically independent molecules in the asymmetric unit of the title compound (I). The molecules form a dimer, in which a pair of N—H···O hydrogen bonds generate an intermolecular *R*_2_^2^(8) ring (Bernstein, *et al.*, 1995). The cyclohexane rings (C9–C14 and C24–C29) in each molecule adopt a chair configuration [the puckering parameters are Q_T_ = 0.547 (5) Å, θ = 180.0 (5) °, φ = 286 (35) ° and Q_T_ = 0.561 (6) Å, θ = 3.7 (5) °, φ = 161 (8) °, respectively] (Cremer & Pople, 1975). In the crystal, molecules are further linked by intermolecular O—H···O hydrogen bonds to form a two-dimensional network parallel to (012). Additional stabilization is provided by weak intermoleclar C—H···O hydrogen bonds (Fig. 2).

### Experimental

4-Methoxybenzene sulfonyl chloride (1.32 g, 6.36 mmol) was added to a solution of tranexamic acid (1.0 g, 6.36 mmol) in distilled water (10 ml). The reaction mixture was stirred at room temperature at pH 8–9, which was adjusted by 1*M* sodium carbonate solution. After completion of the reaction which was observed by the consumption of 4-methoxybenzene sulfonyl chloride, the pH was adjusted at 2–3 using 1 N HCl solution, which resulted in the formation of precipiates. these were filtered off and dried. The product was recrystallized using methanol.

### Refinement

The H atoms of the –NH and –OH groups were located in a difference Fourier map and their positional parameters were restrained [N1—H1N = 0.83 (4), N2—H2N = 0.85 (3), O4—H4O = 0.83 (5) and O10—H10O = 0.82 (7) Å]. Their displacement parameters were refined using a riding model, with *U*_iso_(H) = 1.2*U*_eq_(N) or 1.5*U*_eq_(O). The other H atoms were placed in ideal positions and refined as riding with C—H = 0.93 - 0.98 Å, and *U*_iso_ = 1.2*U*_eq_(C) for CH and CH_2_ groups and *U*_iso_ = 1.5*U*_eq_(C) for CH_3_ group.

### Crystal data

**Table:**

C_15_H_21_NO_5_S	*Z* = 4
*M**_r_* = 327.40	*F*(000) = 696
Triclinic, *P*1	*D*_x_ = 1.353 Mg m^−^^3^
Hall symbol: -P 1	Mo *K*α radiation, λ = 0.71073 Å
*a* = 5.9119 (3) Å	Cell parameters from 4861 reflections
*b* = 10.7223 (7) Å	θ = 2.8–24.1°
*c* = 26.6453 (15) Å	µ = 0.22 mm^−^^1^
α = 79.736 (3)°	*T* = 296 K
β = 89.813 (3)°	Block, colourless
γ = 75.518 (3)°	0.25 × 0.19 × 0.14 mm
*V* = 1607.67 (16) Å^3^	

### Data collection

**Table:**

Bruker APEXII CCD diffractometer	3973 reflections with *I* > 2σ(*I*)
Radiation source: sealed tube	*R*_int_ = 0.048
graphite	θ_max_ = 25.5°, θ_min_ = 0.8°
φ and ω scans	*h* = −7→7
25440 measured reflections	*k* = −12→12
5942 independent reflections	*l* = −32→31

### Refinement

**Table:**

Refinement on *F*^2^	Primary atom site location: structure-invariant direct methods
Least-squares matrix: full	Secondary atom site location: difference Fourier map
*R*[*F*^2^ > 2σ(*F*^2^)] = 0.070	Hydrogen site location: inferred from neighbouring sites
*wR*(*F*^2^) = 0.218	H atoms treated by a mixture of independent and constrained refinement
*S* = 1.03	*w* = 1/[σ^2^(*F*_o_^2^) + (0.1117*P*)^2^ + 1.5531*P*] where *P* = (*F*_o_^2^ + 2*F*_c_^2^)/3
5942 reflections	(Δ/σ)_max_ = 0.001
412 parameters	Δρ_max_ = 0.92 e Å^−^^3^
5 restraints	Δρ_min_ = −0.36 e Å^−^^3^

### Special details

**Table:**

Geometry. Bond distances, angles *etc*. have been calculated using the rounded fractional coordinates. All su's are estimated from the variances of the (full) variance-covariance matrix. The cell e.s.d.'s are taken into account in the estimation of distances, angles and torsion angles
Refinement. Refinement on *F*^2^ for ALL reflections except those flagged by the user for potential systematic errors. Weighted *R*-factors *wR* and all goodnesses of fit *S* are based on *F*^2^, conventional *R*-factors *R* are based on *F*, with *F* set to zero for negative *F*^2^. The observed criterion of *F*^2^ > σ(*F*^2^) is used only for calculating -*R*-factor-obs *etc*. and is not relevant to the choice of reflections for refinement. *R*-factors based on *F*^2^ are statistically about twice as large as those based on *F*, and *R*-factors based on ALL data will be even larger.

### Fractional atomic coordinates and isotropic or equivalent isotropic displacement parameters (Å^2^)

**Table:**

	*x*	*y*	*z*	*U*_iso_*/*U*_eq_	
S1	0.24030 (15)	0.81008 (9)	0.71667 (3)	0.0404 (3)	
O1	0.8071 (6)	0.9302 (4)	0.54159 (12)	0.0852 (16)	
O2	0.1613 (5)	0.6977 (3)	0.71162 (10)	0.0541 (10)	
O3	0.0718 (4)	0.9282 (3)	0.72214 (10)	0.0522 (9)	
O4	1.4245 (6)	0.4189 (3)	0.95284 (13)	0.0772 (12)	
O5	1.2465 (6)	0.6045 (3)	0.97576 (13)	0.0827 (12)	
N1	0.4128 (5)	0.7734 (3)	0.76697 (11)	0.0405 (10)	
C1	0.8230 (11)	0.8647 (6)	0.49907 (19)	0.091 (2)	
C2	0.6699 (8)	0.8966 (4)	0.58007 (16)	0.0593 (16)	
C3	0.6843 (8)	0.9522 (5)	0.62271 (17)	0.0662 (17)	
C4	0.5527 (7)	0.9266 (4)	0.66397 (16)	0.0592 (16)	
C5	0.4061 (6)	0.8452 (3)	0.66319 (13)	0.0402 (11)	
C6	0.3904 (8)	0.7915 (4)	0.62065 (15)	0.0570 (16)	
C7	0.5216 (9)	0.8176 (4)	0.57891 (16)	0.0655 (16)	
C8	0.6083 (6)	0.6550 (4)	0.77250 (14)	0.0446 (12)	
C9	0.7785 (7)	0.6474 (4)	0.81491 (16)	0.0543 (12)	
C10	0.6831 (8)	0.6655 (5)	0.86445 (17)	0.0713 (19)	
C11	0.8758 (8)	0.6601 (5)	0.90438 (17)	0.0722 (19)	
C12	1.0675 (8)	0.5335 (5)	0.90841 (17)	0.0661 (17)	
C13	1.1619 (8)	0.5154 (5)	0.85893 (18)	0.0789 (19)	
C14	0.9709 (8)	0.5220 (4)	0.81929 (17)	0.0649 (17)	
C15	1.2557 (7)	0.5231 (4)	0.94865 (16)	0.0536 (14)	
S2	0.58540 (16)	0.13448 (10)	0.78880 (4)	0.0452 (3)	
O6	1.2525 (6)	0.0555 (4)	0.96080 (12)	0.0810 (14)	
O7	0.3919 (4)	0.2420 (3)	0.79281 (11)	0.0585 (10)	
O8	0.5465 (5)	0.0094 (3)	0.78645 (11)	0.0614 (11)	
O9	1.2935 (9)	0.5689 (4)	0.53819 (18)	0.126 (2)	
O10	1.4743 (11)	0.3683 (4)	0.5428 (2)	0.141 (2)	
N2	0.7154 (5)	0.1743 (3)	0.73693 (11)	0.0445 (10)	
C16	1.2095 (10)	0.1349 (5)	0.99941 (19)	0.0815 (19)	
C17	1.0888 (8)	0.0813 (4)	0.92229 (15)	0.0560 (16)	
C18	1.1491 (8)	0.0089 (5)	0.88368 (18)	0.0702 (16)	
C19	0.9954 (7)	0.0252 (5)	0.84341 (16)	0.0650 (16)	
C20	0.7813 (6)	0.1140 (4)	0.84073 (13)	0.0416 (11)	
C21	0.7221 (7)	0.1857 (4)	0.87863 (15)	0.0564 (14)	
C22	0.8754 (8)	0.1698 (4)	0.91948 (16)	0.0626 (16)	
C23	0.7777 (8)	0.3001 (4)	0.72825 (15)	0.0540 (14)	
C24	0.8709 (7)	0.3303 (4)	0.67579 (15)	0.0520 (12)	
C25	1.1018 (9)	0.2412 (5)	0.6690 (2)	0.0782 (17)	
C26	1.1972 (10)	0.2776 (5)	0.6166 (2)	0.088 (2)	
C27	1.2156 (8)	0.4178 (4)	0.60792 (17)	0.0626 (17)	
C28	0.9834 (9)	0.5085 (5)	0.6117 (2)	0.0756 (19)	
C29	0.8827 (10)	0.4715 (5)	0.6639 (2)	0.079 (2)	
C30	1.3310 (8)	0.4554 (5)	0.55920 (18)	0.0623 (17)	
H1A	0.89090	0.77270	0.51030	0.1370*	
H1B	0.91940	0.89960	0.47420	0.1370*	
H1C	0.66950	0.87770	0.48410	0.1370*	
H1N	0.441 (7)	0.843 (3)	0.7708 (15)	0.0490*	
H3	0.78340	1.00700	0.62340	0.0790*	
H4	0.56250	0.96420	0.69250	0.0710*	
H4O	1.522 (8)	0.425 (6)	0.9742 (19)	0.1160*	
H6	0.29060	0.73700	0.61990	0.0680*	
H7	0.50910	0.78150	0.55010	0.0780*	
H8A	0.68910	0.65460	0.74080	0.0540*	
H8B	0.54670	0.57810	0.77890	0.0540*	
H9	0.85350	0.71910	0.80410	0.0660*	
H10A	0.60360	0.59760	0.87650	0.0860*	
H10B	0.56900	0.74940	0.86060	0.0860*	
H11A	0.94250	0.73440	0.89460	0.0870*	
H11B	0.80800	0.66540	0.93740	0.0870*	
H12	0.99390	0.46160	0.91990	0.0790*	
H13A	1.27480	0.43100	0.86270	0.0950*	
H13B	1.24290	0.58260	0.84700	0.0950*	
H14A	1.03930	0.51690	0.78630	0.0780*	
H14B	0.90450	0.44750	0.82880	0.0780*	
H2N	0.825 (5)	0.109 (3)	0.7335 (15)	0.0530*	
H10O	1.566 (11)	0.400 (4)	0.525 (3)	0.2110*	
H16A	1.20210	0.22420	0.98440	0.1220*	
H16B	1.33380	0.10380	1.02520	0.1220*	
H16C	1.06370	0.12990	1.01460	0.1220*	
H18	1.29440	−0.05080	0.88530	0.0840*	
H19	1.03580	−0.02390	0.81780	0.0780*	
H21	0.57700	0.24570	0.87680	0.0680*	
H22	0.83410	0.21910	0.94500	0.0750*	
H23A	0.89520	0.29810	0.75390	0.0640*	
H23B	0.64060	0.36920	0.73200	0.0640*	
H24	0.75900	0.31900	0.65100	0.0620*	
H25A	1.08620	0.15200	0.67340	0.0940*	
H25B	1.21260	0.24450	0.69520	0.0940*	
H26A	1.35020	0.21950	0.61440	0.1050*	
H26B	1.09420	0.26680	0.59030	0.1050*	
H27	1.31590	0.42480	0.63590	0.0750*	
H28A	0.87730	0.50410	0.58470	0.0910*	
H28B	0.99900	0.59770	0.60730	0.0910*	
H29A	0.97890	0.48750	0.69030	0.0950*	
H29B	0.72660	0.52770	0.66470	0.0950*	

### Atomic displacement parameters (Å^2^)

**Table:**

	*U*^11^	*U*^22^	*U*^33^	*U*^12^	*U*^13^	*U*^23^
S1	0.0355 (5)	0.0484 (6)	0.0369 (5)	−0.0078 (4)	−0.0020 (4)	−0.0110 (4)
O1	0.100 (3)	0.109 (3)	0.055 (2)	−0.039 (2)	0.0312 (18)	−0.0198 (19)
O2	0.0573 (16)	0.0628 (18)	0.0505 (16)	−0.0279 (14)	−0.0009 (13)	−0.0137 (13)
O3	0.0406 (14)	0.0578 (17)	0.0509 (16)	0.0026 (12)	−0.0029 (12)	−0.0122 (13)
O4	0.067 (2)	0.077 (2)	0.077 (2)	0.0080 (18)	−0.0345 (17)	−0.0229 (18)
O5	0.074 (2)	0.086 (2)	0.081 (2)	0.0099 (18)	−0.0353 (18)	−0.039 (2)
N1	0.0417 (16)	0.0421 (18)	0.0370 (16)	−0.0065 (14)	−0.0031 (13)	−0.0113 (14)
C1	0.117 (4)	0.097 (4)	0.053 (3)	−0.015 (3)	0.032 (3)	−0.015 (3)
C2	0.061 (3)	0.065 (3)	0.046 (2)	−0.008 (2)	0.010 (2)	−0.006 (2)
C3	0.068 (3)	0.090 (3)	0.051 (3)	−0.037 (3)	0.007 (2)	−0.016 (2)
C4	0.062 (3)	0.081 (3)	0.045 (2)	−0.030 (2)	0.0037 (19)	−0.022 (2)
C5	0.0376 (18)	0.045 (2)	0.0357 (19)	−0.0050 (16)	−0.0032 (15)	−0.0091 (15)
C6	0.074 (3)	0.062 (3)	0.042 (2)	−0.025 (2)	0.004 (2)	−0.0172 (19)
C7	0.092 (3)	0.069 (3)	0.042 (2)	−0.025 (3)	0.008 (2)	−0.021 (2)
C8	0.044 (2)	0.048 (2)	0.038 (2)	−0.0042 (17)	−0.0018 (16)	−0.0088 (16)
C9	0.053 (2)	0.055 (2)	0.052 (2)	−0.0004 (19)	−0.0134 (19)	−0.0212 (19)
C10	0.055 (3)	0.094 (4)	0.058 (3)	0.000 (2)	−0.005 (2)	−0.023 (3)
C11	0.064 (3)	0.091 (4)	0.050 (3)	0.014 (2)	−0.018 (2)	−0.030 (2)
C12	0.064 (3)	0.072 (3)	0.052 (3)	0.003 (2)	−0.017 (2)	−0.013 (2)
C13	0.061 (3)	0.095 (4)	0.068 (3)	0.013 (3)	−0.011 (2)	−0.028 (3)
C14	0.062 (3)	0.068 (3)	0.056 (3)	0.010 (2)	−0.016 (2)	−0.026 (2)
C15	0.050 (2)	0.060 (3)	0.046 (2)	−0.007 (2)	−0.0110 (18)	−0.0071 (19)
S2	0.0432 (5)	0.0572 (6)	0.0409 (5)	−0.0171 (4)	0.0084 (4)	−0.0179 (4)
O6	0.075 (2)	0.100 (3)	0.062 (2)	0.0009 (19)	−0.0193 (17)	−0.0318 (19)
O7	0.0440 (15)	0.075 (2)	0.0540 (17)	−0.0033 (14)	0.0048 (12)	−0.0222 (14)
O8	0.0729 (19)	0.069 (2)	0.0600 (18)	−0.0405 (16)	0.0159 (15)	−0.0261 (15)
O9	0.156 (4)	0.072 (3)	0.127 (4)	−0.008 (3)	0.088 (3)	0.008 (2)
O10	0.192 (5)	0.078 (3)	0.140 (4)	−0.023 (3)	0.113 (4)	−0.004 (3)
N2	0.0474 (18)	0.0482 (19)	0.0406 (17)	−0.0116 (15)	0.0096 (14)	−0.0159 (14)
C16	0.101 (4)	0.076 (3)	0.065 (3)	−0.014 (3)	−0.024 (3)	−0.018 (3)
C17	0.062 (3)	0.061 (3)	0.044 (2)	−0.012 (2)	−0.0043 (19)	−0.0124 (19)
C18	0.051 (2)	0.089 (3)	0.063 (3)	0.009 (2)	0.000 (2)	−0.032 (3)
C19	0.056 (2)	0.087 (3)	0.053 (3)	−0.004 (2)	0.006 (2)	−0.036 (2)
C20	0.0407 (19)	0.048 (2)	0.0372 (19)	−0.0119 (16)	0.0054 (15)	−0.0102 (16)
C21	0.060 (2)	0.055 (3)	0.050 (2)	0.001 (2)	−0.0001 (19)	−0.0207 (19)
C22	0.077 (3)	0.058 (3)	0.051 (2)	−0.003 (2)	−0.002 (2)	−0.025 (2)
C23	0.067 (3)	0.053 (2)	0.048 (2)	−0.020 (2)	0.0138 (19)	−0.0184 (19)
C24	0.058 (2)	0.052 (2)	0.049 (2)	−0.0171 (19)	0.0115 (18)	−0.0132 (18)
C25	0.072 (3)	0.067 (3)	0.077 (3)	−0.001 (2)	0.015 (3)	0.012 (2)
C26	0.105 (4)	0.058 (3)	0.088 (4)	−0.004 (3)	0.055 (3)	−0.004 (3)
C27	0.065 (3)	0.067 (3)	0.058 (3)	−0.024 (2)	0.014 (2)	−0.007 (2)
C28	0.083 (3)	0.056 (3)	0.083 (4)	−0.016 (2)	0.026 (3)	−0.003 (2)
C29	0.100 (4)	0.057 (3)	0.084 (4)	−0.021 (3)	0.042 (3)	−0.023 (3)
C30	0.067 (3)	0.056 (3)	0.065 (3)	−0.018 (2)	0.021 (2)	−0.011 (2)

### Geometric parameters (Å, °)

**Table:**

S1—O2	1.424 (3)	C8—H8B	0.9700
S1—O3	1.432 (3)	C9—H9	0.9800
S1—N1	1.616 (3)	C10—H10A	0.9700
S1—C5	1.766 (4)	C10—H10B	0.9700
S2—O8	1.428 (3)	C11—H11A	0.9700
S2—N2	1.622 (3)	C11—H11B	0.9700
S2—O7	1.426 (3)	C12—H12	0.9800
S2—C20	1.755 (4)	C13—H13A	0.9700
O1—C1	1.427 (7)	C13—H13B	0.9700
O1—C2	1.359 (6)	C14—H14A	0.9700
O4—C15	1.287 (5)	C14—H14B	0.9700
O5—C15	1.220 (5)	C17—C18	1.390 (6)
O4—H4O	0.83 (5)	C17—C22	1.369 (6)
O6—C16	1.432 (6)	C18—C19	1.367 (6)
O6—C17	1.356 (6)	C19—C20	1.374 (6)
O9—C30	1.211 (7)	C20—C21	1.369 (6)
O10—C30	1.236 (7)	C21—C22	1.378 (6)
O10—H10O	0.82 (7)	C23—C24	1.512 (6)
N1—C8	1.473 (5)	C24—C29	1.510 (7)
N1—H1N	0.83 (4)	C24—C25	1.489 (7)
N2—C23	1.465 (5)	C25—C26	1.527 (8)
N2—H2N	0.85 (3)	C26—C27	1.511 (7)
C2—C3	1.386 (6)	C27—C28	1.486 (7)
C2—C7	1.367 (7)	C27—C30	1.503 (7)
C3—C4	1.372 (6)	C28—C29	1.537 (8)
C4—C5	1.378 (5)	C16—H16A	0.9600
C5—C6	1.373 (5)	C16—H16B	0.9600
C6—C7	1.382 (6)	C16—H16C	0.9600
C8—C9	1.493 (6)	C18—H18	0.9300
C9—C10	1.459 (6)	C19—H19	0.9300
C9—C14	1.515 (6)	C21—H21	0.9300
C10—C11	1.545 (7)	C22—H22	0.9300
C11—C12	1.523 (7)	C23—H23A	0.9700
C12—C13	1.456 (7)	C23—H23B	0.9700
C12—C15	1.518 (6)	C24—H24	0.9800
C13—C14	1.529 (7)	C25—H25A	0.9700
C1—H1B	0.9600	C25—H25B	0.9700
C1—H1C	0.9600	C26—H26A	0.9700
C1—H1A	0.9600	C26—H26B	0.9700
C3—H3	0.9300	C27—H27	0.9800
C4—H4	0.9300	C28—H28A	0.9700
C6—H6	0.9300	C28—H28B	0.9700
C7—H7	0.9300	C29—H29A	0.9700
C8—H8A	0.9700	C29—H29B	0.9700
			
S2···H25B^i^	3.1900	H4O···C15^iii^	2.66 (5)
O3···N2^ii^	3.024 (4)	H4O···O5^iii^	1.84 (5)
O4···O5^iii^	2.660 (5)	H6···O2	2.5500
O5···O4^iii^	2.660 (5)	H7···C1	2.5400
O5···C15^iii^	3.411 (6)	H7···H1C	2.2100
O6···C16^iv^	3.378 (7)	H7···H1A	2.4800
O8···N1^v^	2.961 (4)	H8A···C5	2.8400
O9···O10^vi^	2.631 (8)	H8A···H14A	2.4100
O9···C30^vi^	3.412 (7)	H8B···H13B^i^	2.5500
O10···C30^vi^	3.394 (7)	H8B···H14B	2.4700
O10···O9^vi^	2.631 (8)	H8B···O2	2.7900
O1···H1B^vii^	2.7100	H9···H13B	2.5500
O2···H29A^i^	2.8700	H9···H1N	2.5400
O2···H6	2.5500	H9···H11A	2.5100
O2···H14A^i^	2.7500	H10A···H13B^i^	2.3300
O2···H8B	2.7900	H10A···H14B	2.5800
O3···H19^ii^	2.6900	H10A···H12	2.5500
O3···H25A^ii^	2.5400	H10A···C13^i^	3.0200
O3···H2N^ii^	2.18 (3)	H10A···C15^i^	2.9700
O4···H16A	2.7400	H10B···H1N	2.4700
O4···H4O^iii^	2.85 (6)	H10B···N1	2.6100
O4···H22^viii^	2.8400	H10B···H18^ii^	2.5300
O4···H13A	2.5300	H10O···O9^vi^	1.89 (8)
O5···H11B	2.6700	H10O···H10O^vi^	2.27 (8)
O5···H11A	2.7500	H10O···C30^vi^	2.65 (7)
O5···H4O^iii^	1.84 (5)	H10O···O10^vi^	2.76 (6)
O6···H16B^iv^	2.5900	H11A···H13B	2.5900
O7···H21	2.5100	H11A···H9	2.5100
O7···H25B^i^	2.8000	H11A···O5	2.7500
O7···H23B	2.6200	H11B···O5	2.6700
O8···H1N^v^	2.14 (3)	H11B···C16^xiii^	2.9300
O8···H4^v^	2.6300	H11B···H16A^xiii^	2.5700
O9···H28B	2.5400	H12···H14B	2.5200
O9···H1A	2.8000	H12···H10A	2.5500
O9···H10O^vi^	1.89 (8)	H13A···H21^viii^	2.2900
O10···H26A	2.4900	H13A···O4	2.5300
O10···H10O^vi^	2.76 (6)	H13B···H8B^viii^	2.5500
N1···O8^ix^	2.961 (4)	H13B···C8^viii^	3.0800
N2···O3^x^	3.024 (4)	H13B···C10^viii^	3.0200
N1···H10B	2.6100	H13B···H11A	2.5900
N1···H4	2.8900	H13B···H9	2.5500
N2···H25A	2.7500	H13B···H10A^viii^	2.3300
C15···O5^iii^	3.411 (6)	H14A···H8A	2.4100
C16···O6^iv^	3.378 (7)	H14A···O2^viii^	2.7500
C30···O10^vi^	3.394 (7)	H14B···H8B	2.4700
C30···O9^vi^	3.412 (7)	H14B···H10A	2.5800
C1···H26B^xi^	2.9600	H14B···H12	2.5200
C1···H7	2.5400	H16A···O4	2.7400
C2···H1C^xii^	2.9900	H16A···C22	2.8300
C3···H26A^ii^	3.0200	H16A···H22	2.4400
C4···H1N	2.95 (4)	H16A···H11B^xiii^	2.5700
C5···H8A	2.8400	H16B···O6^iv^	2.5900
C6···H29B	3.0600	H16C···C22	2.6900
C7···H1C	2.6900	H16C···H22	2.2200
C7···H1A	2.8500	H16C···C17^xiv^	2.9000
C8···H13B^i^	3.0800	H18···H10B^x^	2.5300
C10···H13B^i^	3.0200	H19···O3^x^	2.6900
C10···H1N	2.98 (4)	H19···H2N	2.5700
C13···H10A^viii^	3.0200	H21···O7	2.5100
C15···H10A^viii^	2.9700	H21···H13A^i^	2.2900
C15···H4O^iii^	2.66 (5)	H22···O4^i^	2.8400
C16···H22	2.5300	H22···C16	2.5300
C16···H11B^xiii^	2.9300	H22···H16A	2.4400
C17···H16C^xiv^	2.9000	H22···H16C	2.2200
C19···H2N	3.01 (4)	H23A···C20	2.9500
C20···H23A	2.9500	H23A···H25B	2.4600
C22···H16C	2.6900	H23A···H29A	2.5500
C22···H16A	2.8300	H23B···O7	2.6200
C25···H2N	2.81 (3)	H23B···H29B	2.3900
C30···H10O^vi^	2.65 (7)	H24···H26B	2.5700
H1A···O9	2.8000	H24···H27^i^	2.5800
H1A···C7	2.8500	H25A···O3^x^	2.5400
H1A···H7	2.4800	H25A···N2	2.7500
H1B···O1^vii^	2.7100	H25A···H2N	2.2900
H1C···C7	2.6900	H25B···S2^viii^	3.1900
H1C···H7	2.2100	H25B···O7^viii^	2.8000
H1C···C2^xii^	2.9900	H25B···H23A	2.4600
H1N···O8^ix^	2.14 (3)	H25B···H27	2.4700
H1N···C4	2.95 (4)	H26A···O10	2.4900
H1N···C10	2.98 (4)	H26A···C3^x^	3.0200
H1N···H4	2.4600	H26B···H24	2.5700
H1N···H9	2.5400	H26B···H28A	2.5200
H1N···H10B	2.4700	H26B···C1^xi^	2.9600
H2N···C25	2.81 (3)	H27···H24^viii^	2.5800
H2N···O3^x^	2.18 (3)	H27···H25B	2.4700
H2N···C19	3.01 (4)	H27···H29A	2.4900
H2N···H19	2.5700	H28A···H26B	2.5200
H2N···H25A	2.2900	H28B···O9	2.5400
H4···O8^ix^	2.6300	H29A···O2^viii^	2.8700
H4···N1	2.8900	H29A···H23A	2.5500
H4···H1N	2.4600	H29A···H27	2.4900
H4O···O4^iii^	2.85 (6)	H29B···C6	3.0600
H4O···H4O^iii^	2.26 (8)	H29B···H23B	2.3900
			
O2—S1—O3	119.03 (18)	C13—C12—H12	107.00
O2—S1—N1	108.36 (17)	C12—C13—H13B	109.00
O2—S1—C5	107.61 (16)	C12—C13—H13A	109.00
O3—S1—N1	105.26 (16)	C14—C13—H13B	109.00
O3—S1—C5	108.38 (16)	H13A—C13—H13B	108.00
N1—S1—C5	107.74 (16)	C14—C13—H13A	109.00
O8—S2—C20	107.74 (19)	C9—C14—H14A	109.00
N2—S2—C20	107.95 (17)	H14A—C14—H14B	108.00
O7—S2—N2	107.77 (17)	C13—C14—H14A	109.00
O7—S2—C20	107.64 (18)	C13—C14—H14B	109.00
O7—S2—O8	119.76 (18)	C9—C14—H14B	109.00
O8—S2—N2	105.49 (17)	O6—C17—C18	115.2 (4)
C1—O1—C2	117.8 (4)	O6—C17—C22	125.2 (4)
C15—O4—H4O	107 (4)	C18—C17—C22	119.6 (4)
C16—O6—C17	117.8 (4)	C17—C18—C19	120.2 (5)
C30—O10—H10O	110 (3)	C18—C19—C20	120.1 (4)
S1—N1—C8	118.3 (2)	S2—C20—C19	119.8 (3)
S1—N1—H1N	106 (3)	S2—C20—C21	120.5 (3)
C8—N1—H1N	118 (3)	C19—C20—C21	119.8 (4)
S2—N2—C23	118.3 (3)	C20—C21—C22	120.6 (4)
C23—N2—H2N	115 (2)	C17—C22—C21	119.8 (4)
S2—N2—H2N	108 (3)	N2—C23—C24	111.9 (3)
C3—C2—C7	119.8 (4)	C25—C24—C29	110.6 (4)
O1—C2—C7	125.5 (4)	C23—C24—C29	110.2 (4)
O1—C2—C3	114.7 (4)	C23—C24—C25	113.5 (4)
C2—C3—C4	120.2 (4)	C24—C25—C26	112.4 (4)
C3—C4—C5	120.1 (4)	C25—C26—C27	110.4 (4)
C4—C5—C6	119.6 (4)	C26—C27—C28	110.6 (4)
S1—C5—C4	120.1 (3)	C26—C27—C30	112.9 (4)
S1—C5—C6	120.3 (3)	C28—C27—C30	112.6 (4)
C5—C6—C7	120.6 (4)	C27—C28—C29	110.3 (4)
C2—C7—C6	119.8 (4)	C24—C29—C28	113.3 (4)
N1—C8—C9	112.4 (3)	O9—C30—O10	121.1 (5)
C10—C9—C14	111.5 (4)	O9—C30—C27	120.7 (5)
C8—C9—C14	109.5 (4)	O10—C30—C27	118.1 (5)
C8—C9—C10	117.0 (4)	O6—C16—H16A	110.00
C9—C10—C11	111.9 (4)	O6—C16—H16B	109.00
C10—C11—C12	110.6 (4)	O6—C16—H16C	109.00
C11—C12—C13	111.7 (4)	H16A—C16—H16B	109.00
C13—C12—C15	112.8 (4)	H16A—C16—H16C	109.00
C11—C12—C15	111.9 (4)	H16B—C16—H16C	109.00
C12—C13—C14	112.1 (4)	C17—C18—H18	120.00
C9—C14—C13	111.6 (4)	C19—C18—H18	120.00
O4—C15—O5	122.4 (4)	C18—C19—H19	120.00
O4—C15—C12	114.3 (4)	C20—C19—H19	120.00
O5—C15—C12	123.3 (4)	C20—C21—H21	120.00
H1A—C1—H1B	109.00	C22—C21—H21	120.00
H1A—C1—H1C	109.00	C17—C22—H22	120.00
H1B—C1—H1C	110.00	C21—C22—H22	120.00
O1—C1—H1B	109.00	N2—C23—H23A	109.00
O1—C1—H1C	109.00	N2—C23—H23B	109.00
O1—C1—H1A	110.00	C24—C23—H23A	109.00
C4—C3—H3	120.00	C24—C23—H23B	109.00
C2—C3—H3	120.00	H23A—C23—H23B	108.00
C5—C4—H4	120.00	C23—C24—H24	107.00
C3—C4—H4	120.00	C25—C24—H24	107.00
C7—C6—H6	120.00	C29—C24—H24	107.00
C5—C6—H6	120.00	C24—C25—H25A	109.00
C2—C7—H7	120.00	C24—C25—H25B	109.00
C6—C7—H7	120.00	C26—C25—H25A	109.00
C9—C8—H8A	109.00	C26—C25—H25B	109.00
C9—C8—H8B	109.00	H25A—C25—H25B	108.00
H8A—C8—H8B	108.00	C25—C26—H26A	110.00
N1—C8—H8B	109.00	C25—C26—H26B	110.00
N1—C8—H8A	109.00	C27—C26—H26A	110.00
C10—C9—H9	106.00	C27—C26—H26B	110.00
C14—C9—H9	106.00	H26A—C26—H26B	108.00
C8—C9—H9	106.00	C26—C27—H27	107.00
C9—C10—H10B	109.00	C28—C27—H27	107.00
C11—C10—H10A	109.00	C30—C27—H27	107.00
C11—C10—H10B	109.00	C27—C28—H28A	110.00
C9—C10—H10A	109.00	C27—C28—H28B	110.00
H10A—C10—H10B	108.00	C29—C28—H28A	110.00
C12—C11—H11A	110.00	C29—C28—H28B	110.00
C12—C11—H11B	110.00	H28A—C28—H28B	108.00
H11A—C11—H11B	108.00	C24—C29—H29A	109.00
C10—C11—H11A	110.00	C24—C29—H29B	109.00
C10—C11—H11B	110.00	C28—C29—H29A	109.00
C15—C12—H12	107.00	C28—C29—H29B	109.00
C11—C12—H12	107.00	H29A—C29—H29B	108.00
			
O2—S1—N1—C8	51.9 (3)	C10—C9—C14—C13	−54.1 (5)
O3—S1—N1—C8	−179.8 (3)	C8—C9—C14—C13	174.8 (4)
C5—S1—N1—C8	−64.3 (3)	C9—C10—C11—C12	−54.7 (5)
O2—S1—C5—C4	−163.4 (3)	C10—C11—C12—C13	54.4 (5)
O2—S1—C5—C6	16.4 (4)	C10—C11—C12—C15	−178.1 (4)
O3—S1—C5—C4	66.7 (3)	C13—C12—C15—O5	130.0 (5)
O3—S1—C5—C6	−113.5 (3)	C15—C12—C13—C14	178.3 (4)
N1—S1—C5—C4	−46.8 (3)	C13—C12—C15—O4	−51.7 (6)
N1—S1—C5—C6	133.1 (3)	C11—C12—C13—C14	−54.7 (6)
C20—S2—N2—C23	−65.3 (3)	C11—C12—C15—O4	−178.7 (4)
O7—S2—C20—C19	−173.3 (3)	C11—C12—C15—O5	3.0 (6)
O7—S2—C20—C21	6.8 (4)	C12—C13—C14—C9	54.2 (5)
O8—S2—C20—C19	56.2 (4)	O6—C17—C18—C19	178.8 (4)
O8—S2—C20—C21	−123.7 (3)	C22—C17—C18—C19	−0.6 (7)
N2—S2—C20—C19	−57.3 (4)	O6—C17—C22—C21	−178.9 (4)
N2—S2—C20—C21	122.9 (3)	C18—C17—C22—C21	0.4 (7)
O7—S2—N2—C23	50.7 (3)	C17—C18—C19—C20	0.5 (7)
O8—S2—N2—C23	179.7 (3)	C18—C19—C20—S2	179.9 (4)
C1—O1—C2—C3	172.6 (4)	C18—C19—C20—C21	−0.3 (7)
C1—O1—C2—C7	−8.9 (7)	S2—C20—C21—C22	180.0 (3)
C16—O6—C17—C18	174.6 (4)	C19—C20—C21—C22	0.1 (6)
C16—O6—C17—C22	−6.1 (7)	C20—C21—C22—C17	−0.1 (7)
S1—N1—C8—C9	168.8 (3)	N2—C23—C24—C25	−67.8 (5)
S2—N2—C23—C24	−173.2 (3)	N2—C23—C24—C29	167.6 (4)
O1—C2—C3—C4	179.5 (4)	C23—C24—C25—C26	−177.4 (4)
C7—C2—C3—C4	0.9 (7)	C29—C24—C25—C26	−52.9 (5)
O1—C2—C7—C6	−179.7 (4)	C23—C24—C29—C28	178.2 (4)
C3—C2—C7—C6	−1.3 (7)	C25—C24—C29—C28	51.9 (6)
C2—C3—C4—C5	0.2 (7)	C24—C25—C26—C27	56.8 (6)
C3—C4—C5—C6	−0.9 (6)	C25—C26—C27—C28	−58.6 (6)
C3—C4—C5—S1	179.0 (3)	C25—C26—C27—C30	174.2 (4)
S1—C5—C6—C7	−179.3 (3)	C26—C27—C28—C29	57.1 (5)
C4—C5—C6—C7	0.5 (6)	C30—C27—C28—C29	−175.6 (4)
C5—C6—C7—C2	0.6 (7)	C26—C27—C30—O9	155.7 (5)
N1—C8—C9—C10	50.4 (5)	C26—C27—C30—O10	−28.1 (7)
N1—C8—C9—C14	178.6 (3)	C28—C27—C30—O9	29.7 (7)
C8—C9—C10—C11	−178.1 (4)	C28—C27—C30—O10	−154.2 (5)
C14—C9—C10—C11	54.7 (5)	C27—C28—C29—C24	−54.5 (6)

Symmetry codes: (i) *x*−1, *y*, *z*; (ii) *x*−1, *y*+1, *z*; (iii) −*x*+3, −*y*+1, −*z*+2; (iv) −*x*+3, −*y*, −*z*+2; (v) *x*, *y*−1, *z*; (vi) −*x*+3, −*y*+1, −*z*+1; (vii) −*x*+2, −*y*+2, −*z*+1; (viii) *x*+1, *y*, *z*; (ix) *x*, *y*+1, *z*; (x) *x*+1, *y*−1, *z*; (xi) −*x*+2, −*y*+1, −*z*+1; (xii) −*x*+1, −*y*+2, −*z*+1; (xiii) −*x*+2, −*y*+1, −*z*+2; (xiv) −*x*+2, −*y*, −*z*+2.

### Hydrogen-bond geometry (Å, °)

**Table:**

*D*—H···*A*	*D*—H	H···*A*	*D*···*A*	*D*—H···*A*
N1—H1N···O8^ix^	0.83 (4)	2.14 (3)	2.961 (4)	173 (3)
N2—H2N···O3^x^	0.85 (3)	2.18 (3)	3.024 (4)	172 (3)
O4—H4O···O5^iii^	0.83 (5)	1.84 (5)	2.660 (5)	166 (6)
O10—H10O···O9^vi^	0.82 (7)	1.89 (8)	2.631 (8)	151 (7)
C6—H6···O2	0.93	2.55	2.918 (5)	104
C10—H10B···N1	0.97	2.61	2.945 (5)	100
C16—H16B···O6^iv^	0.96	2.59	3.378 (7)	139
C21—H21···O7	0.93	2.51	2.890 (5)	105
C25—H25A···O3^x^	0.97	2.54	3.450 (6)	157

Symmetry codes: (ix) *x*, *y*+1, *z*; (x) *x*+1, *y*−1, *z*; (iii) −*x*+3, −*y*+1, −*z*+2; (vi) −*x*+3, −*y*+1, −*z*+1; (iv) −*x*+3, −*y*, −*z*+2.
